# Health Determinants among North Americans Experiencing Homelessness and Traumatic Brain Injury: A Scoping Review

**DOI:** 10.1089/neur.2021.0010

**Published:** 2021-08-16

**Authors:** Kaitlin J. Zeiler, Alwyn Gomez, Francois Mathieu, Frederick A. Zeiler

**Affiliations:** ^1^ Undergraduate Psychology Program, Faculty of Arts, University of Manitoba, Winnipeg, Manitoba, Canada.; ^2^ Section of Neurosurgery, Department of Surgery, University of Manitoba, Winnipeg, Manitoba, Canada.; ^3^ Department of Anatomy and Cell Science, Rady Faculty of Health Sciences, University of Manitoba, Winnipeg, Manitoba, Canada.; ^4^ Section of Neurosurgery, Department of Surgery, University of Toronto, Toronto, Ontario, Canada.; ^5^ Biomedical Engineering, Faculty of Engineering, University of Manitoba, Winnipeg, Manitoba, Canada.; ^6^ Division of Anesthesia, Department of Medicine, University of Cambridge, Cambridge, UK.; ^7^ Centre on Aging, University of Manitoba, Winnipeg, Manitoba, Canada.

**Keywords:** determinants of health, homeless, North America, traumatic brain injury

## Abstract

Traumatic brain injury (TBI) in those experiencing homelessness has been described in recent literature as a contributor to increased morbidity, decreased functional independence, and early mortality. In this systematically conducted scoping review, we aimed to better delineate the health determinants—as defined by Health Canada/Centers for Disease Control and Prevention (CDC)—associated with TBI in North Americans experiencing homelessness. BIOSIS, MEDLINE, CINAHL, EMBASE, SCOPUS, and Global Health were searched from inception to December 30, 2020. Gray literature search consisted of relevant meeting proceedings. A two-step process was undertaken, assessing title/abstract and full articles, respectively, based on inclusion/exclusion criteria, leading to the final 20 articles included in the review. Data were abstracted, assessing the aims, literature quality, and bias. Five health determinants displayed strong associations with TBI in those North Americans experiencing homelessness, including male gender, poor physical environment, negative personal health behaviors, adverse childhood experiences (ACEs), and low educational attainment. In those studies displaying a comparator population experiencing homelessness without TBI, the TBI group displayed trends toward increased disparity in Health Canada and CDC defined health determinants. Most studies suffered from moderate limitations. There are associations between male gender, poor physical environment, negative personal health behaviors, ACEs, and limited education in those experiencing homelessness and TBI. The results suggest that those experiencing homelessness with TBI in North America suffer poorer health consequences than those without TBI. Future research on TBI in North Americans experiencing homelessness should focus on health determinants as potential areas for intervention, which may lead to improved outcomes for those experiencing both homelessness and TBI.

## Introduction

Traumatic brain injury (TBI) has been recognized as a major public health issue in many jurisdictions, with approximately 50 million people sustaining a TBI annually worldwide.^1^ The incidence of TBI in the United States is estimated to be 1.7 million and in Canada, 23,000 people are hospitalized due to TBI annually.^2,3^ TBI is defined as “an alteration in brain function, or other evidence of brain pathology, caused by an external force.”^4^ Despite a growing research field and multiplications of public health efforts in recent years, it continues to be the greatest cause of disability and death from trauma in North America.^5^

Regardless of severity, TBI broadly affects all populations and sub-populations. However, certain sub-populations may be overrepresented in the global burden of TBI. Such populations may include, but are not limited to: visible minorities, Indigenous populations, vulnerable populations, and veterans.^6,7^ There has recently been an expansion of TBI-related research to investigate any disparities in the TBI incidence among various sub-populations. Those experiencing homelessness in North America, are one such sub-population of interest.

Homelessness in North America is a major societal problem. Many ambiguous definitions of homelessness are described in the literature, as the circumstances people find themselves in prior to becoming homeless are very different. Homelessness is defined as lacking a permanent residence (living on the street, in a shelter, with family, in substandard housing, etc.) or in threat of losing your residence due to financial difficulty or violence.^8^ Although it is difficult to determine the exact incidence and prevalence of homelessness in North America, in Canada approximately 150,000 to 300,000 people are experiencing homelessness at any point per annum.^9^ The United States has estimated that approximately 2.5–3.5 million individuals experience homelessness every year.^10^ More recent data from the National Alliance to End Homelessness and the U.S. Department of Housing and Urban Development (HUD) indicate that 17 of every 10,000 persons in the United States experienced at least one night of homelessness in 2018, with up to two-thirds being individuals, and the remaining representing family units with children.^11,12^

People experiencing homelessness have been described as suffering more health disparities and as being more vulnerable to harm than the general population.^13^ The co-existing burden of TBI and homelessness has been identified in recent years as a major public health issue. TBI in those experiencing homelessness has been described as a contributor to increased morbidity, decreased functional independence, and early mortality.^14^

Despite the association between TBI and homelessness having been preliminarily examined in North America, it remains unclear which determinants of health underly these associations. The determinants of health as defined by Health Canada and the Centers for Disease Control and Prevention (CDC) are frameworks that describe the influence our environment, social community, and our economic and personal factors have on our health and states of disease.^15,16^ Health Canada has defined a group of factors that contribute to a person's health and well-being described as the determinants of health. These consist of: income and social status, employment and working conditions, education and literacy, childhood experiences, physical environments, social supports and coping skills, healthy behaviors, access to healthcare, biology and genetic endowment, and gender and culture. The CDC defines the social determinants of health for Americans as: economic stability, education, social and community context, health and healthcare, and neighborhood and built environment.

To fully understand the contributing factors related to TBI in those North Americans experiencing homelessness, one must evaluate the related determinants of health. Recent analysis into the North American Indigenous population has supported associations between specific health determinants, as defined by Health Canada and the CDC, with the incidence of a TBI.^7^ This work was conducted by our group with the aim of producing a comprehensive overview of the association between social determinants of health and TBI in North American Indigenous populations. Where Indigenous populations referred to those individuals identifying as First Nations/Indigenous peoples in either Canada or North America. This population is considered a vulnerable population with significant disparities in health in both countries. Within this prior review we identified significant gaps in the existing literature, including difficulties in teasing out data on the sub-population of interest from larger general populations of patients with TBI described in this work. Given the homeless populations in North American are also vulnerable and at higher risk for suffering TBI, we elected to explore the link between social determinants of health and TBI in this population. At the time of the writing of this article similar work had yet to be conducted in the entire population of North Americans experiencing homelessness.

This study aimed to better understand the health determinants linked to TBI in those North Americans experiencing homelessness, by systematically reviewing the available literature on the topic. Available data in support of associations with temporality of TBI in relation to homeless episodes, repeat TBI episodes while experiencing homelessness, and any specific sub-populations of those experiencing homelessness who may be at higher risk are outlined. The goal was not to definitively try to answer which specific health determinants are associated/causal with TBI in those suffering from homelessness, but merely to highlight the heterogeneous and limited literature body on the topic, to spark future directed studies in this area.

## Methods

The following systematically conducted scoping review was conducted in accordance with the techniques described in the *Cochrane Handbook for Systematic Review of Interventions*^17^ and reported in accordance with the Preferred Reporting Items for Systematic Reviews and Meta-Analysis (PRISMA) guidelines.^18^ The PRISMA checklist for this study can be found in Supplementary Appendix S1.

### Research question

The following question was posed: “In those North Americans experiencing homelessness, what are the determinants (as defined by Health Canada and the CDC) that are associated with sustaining a TBI?” For the purpose of this study, homelessness is all encompassing, which includes: absolute homelessness, hidden or concealed homelessness, and relative homelessness.^8^

### Primary and secondary outcomes

The primary outcome of the study is as follows:

- Explore the Health Canada and CDC defined determinants of health as they relate to TBI in those experiencing homelessness in North America.

The following secondary outcomes were also assessed:

- Assess the temporality between the incidence of TBI and experiencing homelessness. This will be explored by assessing the determinants of health associated with suffering a TBI prior to experiencing homelessness and the determinants associated with suffering a TBI while experiencing homelessness.
- The determinants associated with suffering repeat TBI episodes while experiencing homelessness.
- The presence of any sub-populations experiencing homelessness within North America who have been identified as being at higher risk for suffering a TBI. Sub-populations may consist of different ethnic populations and/or regional disparities.

### Search strategy

The health research databases searched included: BIOSIS, MEDLINE, CINAHL, EMBASE, SCOPUS, and Global Health from inception to December 30, 2020. Selected search terms included: “Acquired brain injury,” “Traumatic brain injury,” “Head trauma,” “Cerebral trauma,” “Vulnerable population,” and “Street people.” Supplementary Appendix S2 provides a sample search strategy employed for MEDLINE, with identical search strategies employed for the other electronic databases listed.

Additionally, a gray literature search was conducted, and whereby meeting proceedings for professional societies linked with TBI research were searched for the last 5 years. Finally, the reference sections of relevant primary research articles and review articles were hand searched to avoid missing relevant articles. Supplementary Appendix S3 lists the selected professional societies for which meeting proceedings were searched.

### Inclusion/Exclusion criteria

Studies included in this review satisfied the following criteria: North America Studies (Canada and the United States only), an identified population experiencing homelessness, individuals with a documented TBI (of any severity), persons of any age, English studies only, studies must have listed at least one determinant of health (as defined by Health Canada and/or CDC), and studies reporting only primary research. We included studies with and without non-TBI comparator groups, to provide the most robust review on the topic to date. Such a comprehensive review strategy was deemed important, as the current literature body is very sparce, especially for those studies with comparator groups. Subsequently, to provide a proper baseline understanding of the available literature on the topic of the link between social determinants of health and TBI in the homeless population, such a wide reaching review strategy was employed. It is through such comprehensive understanding of the available literature that future studies may be planned.

Exclusion criteria included: non-North American studies, non-English studies, populations not experiencing homelessness, and no documented TBI in the study population.

### Filtering process

The filtering process took place in a two-step fashion with two independent reviewers. First, all results of the database searches were filtered based on title and abstract, applying the above-defined inclusion/exclusion criteria, keeping only those that met the inclusion characteristics. Second, any results that passed the first filter stage subsequently had the full versions of the article pulled, checking to ensure that they still met the inclusion criteria upon evaluation of the full study. Articles that passed the first and second filter stage were included in the final review. Any discrepancies between the two reviewers about articles to include were solved by a third independent reviewer, as needed.

### Data abstraction

The data were independently extracted by two reviewers into electronic spreadsheets created for the systematically conducted scoping review. The extracted study characteristics included: study design, study location and setting, study population, data analysis, primary and secondary outcomes, and determinants (as defined by Health Canada and CDC). Supplementary Appendix S4 defines the variables of the data extracted (descriptive study characteristics) that included: study design, study setting, study location, data analysis method, population characteristics, primary and secondary outcomes, social determinants as describes by Health Canada and CDC, sample size, rate of TBI in the study, temporal nature of TBI related to experiencing homelessness, associated mental health issues, and any sub-population at risk for TBI identified.

### Assessment of determinants of health

Using the social determinants of health, as defined as Health Canada^16^ and the CDC,^15^ each study was individually assessed for the association between specific determinants and TBI in those experiencing homelessness, extracting and summarizing the information related to the above-defined primary and secondary study outcomes.

### Synthesis of results

The association between Health Canada and CDC defined determinants of health and TBI in those North Americans experiencing homelessness was summarized in tables and a narrative analysis was used within the body of the Results section. Given the heterogeneity of the studies included in the final review, no formal statistical analysis or meta-analysis was conducted.

### Assessment of study validity and bias

Each study was individually assessed for internal validity using the Critical Appraisal Skills Programme (CASP) checklist tool^19^ for observational studies, which provides specific criteria to evaluate and appraise the quality of the included research studies according to domains, as described in the CASP tool applied in Supplementary Appendix S5. Further, full details of the CASP checklist for each individual, included study can also be found tabulated in Supplementary Appendix S5.

## Results

### Search results

**FIG. 1.**
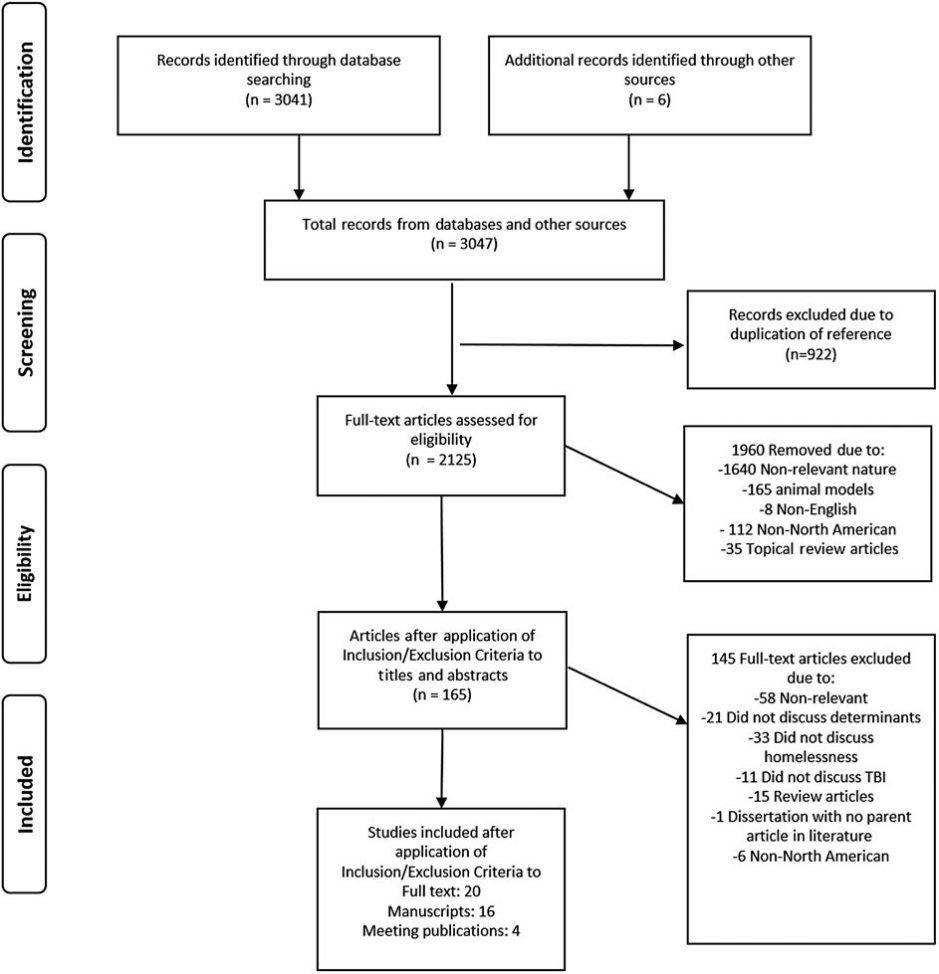
PRISMA flow diagram. PRIMSA, Preferred Reporting in Systematic Reviews and Meta-Analysis.

Following the search of databases, 3041 articles were identified in the results, and the gray literature results revealed 6 articles. This left a total of 3047 articles identified after primary searches of databases and gray literature. After removing duplicates, 2125 articles were left to review for the first filter of results. Following review of the titles and abstracts, 165 met the inclusion/exclusion criteria. Upon reviewing the full articles for eligibility, 20 articles and abstracts met the inclusion/exclusion criteria. Figure 1 displays the PRISMA flow diagram and reasons for exclusion. These final 20 articles were included in the systematically conducted scoping review.

### Study characteristics

In total, the final results included 20 articles in the systematically conducted scoping review. Of the 20 articles included in the review, study designs were as follows: 13 cross-sectional studies, 2 retrospective cohorts, 1 prospective cohort, 3 secondary analysis of randomized controlled trials, and 1 longitudinal study. Individual study details can be seen in Table 1.

**Table 1.** General Study Characteristics and Design for Final included Studies

Author and year	Journal/Abstract	Study design	Setting and context	Age range, years	Geographic location/Participants	Primary/Secondary outcomes
Bacciardi et al., 2017	Journal article	Cross-sectional observational study	At home/Chez Soi Housing first study for mentally ill homeless people across five sites in Canada	19-67	Five major cities in Canada: Moncton, Montreal, Toronto, Winnipeg, and Vancouver. Participants: homeless/precariously housed and presence of a mental illness	Examine association between BD and TBI, comparing homeless affected by either BD, UD, or SSD.
Barnes et al., 2015	Journal article	Cross-sectional participant interviews and retrospective chart review	Veterans seeking homeless services at Veterans Affairs Hospitals (1 eastern USA and 1 western USA)	Mean age: 51.8	USA: 1 eastern hospital, 1 western hospital	Describe the relationship between homelessness and TBIs in the veteran population (secondary analysis of data).
Bymaster et al., 2017	Journal article	Cross-sectional participant surveys	Individuals seeking healthcare at clinic for homeless	21-75	2 clinic sites of Santa Clara County Homeless Program (USA)	Describe the pediatric profile of a homeless person in Santa Clara. Then investigate ACE, TBI, and home breakdown in the lives of the homeless in this location.
Cusimano et al., 2018	Abstract	Cross-sectional participant interviews	NR	NR	Toronto, ON, Canada	Analyze experiences of violence in homeless individuals, the majority of whom have a history of TBI.
Gargaro et al., 2016	Journal article	Cross-sectional participant surveys	Clients seeking assistance from community treatment team for homeless persons with mental illness and/or substance use issues	25-71	Toronto, ON, Canada	Identify whether clients seeking support from ACTT for homeless people also had histories of brain injury, if awareness could be raised among team member to screen clients for brain injury.
Gonzalez et al., 2001	Journal article	Cross- sectional study	Homeless individuals receiving healthcare associated with a shelter and outreach program for homeless people	19-61	Miami, FL, USA	Examine neurological functioning in a homeless population sample and compare an abbreviated battery of tests to the MMSE.
Harris et al., 2015	Abstract	Retrospective cohort study	All females who suffered TBI and were treated at VA Palo Alto Health Care System, Polytrauma System of Care	NR	Palo Alto, CA, USA	Characterization of female TBI/polytrauma patient and factors associated with head injury.
Hwang et al., 2008	Journal article	Cross-sectional survey design: stratified enrollment	Homeless persons who use shelters, meal programs	Mean: 37.4	Toronto, ON, Canada	Determine lifetime prevalence of TBI in a sample of homeless individuals. To identify temporal relationships between TBI and onset of homelessness. Characterize association of health issues and TBI in the homeless.
Kozloff et al., 2016	Journal article	Secondary analysis of randomized controlled trial	Homeless individuals with a mental disorder, participating in a housing first trial in five cities in Canada	Mean: 21.6 (<24 years cohort)	Five cities across Canada: Moncton, Montreal, Toronto, Winnipeg, and Vancouver	Compared demographic, clinical characteristics, and service use of youth and young adults with mental illness who are homeless.
Mackelprang et al., 2014	Journal article	Cross-sectional survey	Homeless individuals recruited from locations that provide services to the homeless	11-28	St. Paul, MN, USA.	Determine prevalence of TBI in youth and young adults who are homeless. Examine difference of sociodemographic characteristics, mental health, exposure to violence, and performance of ADLs of TBI group versus control.
Mejia-Lancheros et al., 2020	Journal article	Secondary analysis of randomized controlled trial	Homeless individuals with a mental health disorder participating in housing first trial.	Mean: 40.6 (SD-11.7)	Toronto, ON, Canada	Determine the effect of the housing first intervention on incidents of physical violence related TBI. Determine the number of physical violence related TBI events in the study group.
Nikoo et al., 2017	Journal article	Longitudinal study	Homeless and vulnerably housed individuals in three Canadian cities	Mean (baseline): 42.2	Three cities across Canada: Vancouver, Ottawa, and Toronto	Determine risk factors for incidence TBI among homeless and vulnerably housed individuals in a housing transition study (secondary analysis).
Panenka et al., 2015	Abstract	Cross-sectional study	Individuals living in marginal housing (SRO hotel rooms)	NR	Vancouver, BC, Canada	Determine prevalence and severity of TBI in cohort people living in city's SRO hotels. Outline the relationships of TBI and health variables.
Solliday-McRoy et al., 2004	Journal article	Cross-sectional study	Homeless men living in a shelter	20-63	Milwaukee, WI, USA	Assess the neuropsychological functioning in homeless men living in a shelter.
Song et al., 2018	Journal article	Cross-sectional survey	Homeless individuals in three cities in Canada	Mean: non-TBI cohort, 37.5; TBI cohort, 38.1	Three cities in BC, Canada: Vancouver, Victoria, and Prince George	Examine association of childhood trauma and TBI in a homeless population. Explore associations of different types and number of traumas experienced and TBI.
Svoboda et al., 2014	Journal article	Retrospective cohort study (sub-analysis of larger study)	Shelter for men who are chronically homeless with severe alcohol problems. Control groups: hostel for men in general homeless population and LIH housing sites	Mean: 43.7	Toronto, ON, Canada	Examining the incidence of HI to ED among cohorts of LIH, homeless, and chronically homeless alcohol dependent men. Examination and temporal pattern of HI and CSH in these vulnerable populations.
Topolovec-Vranic et al., 2014	Journal article	Observational study (three cohorts of men): cross-sectional study	Men's shelter hostel program, harm reduction program, and long-term care program	Mean: 54.2	Toronto, ON, Canada	Determine prevalence, temporality relationship of TBI and homelessness, and mechanisms of injury among a sample of homeless men. Associated characteristics of men with TBI vs. no TBI.
Topolovec-Vranic et al., 2017	Journal article	Secondary analysis of randomized controlled trial	Homeless individuals with a mental illness, participating in a housing trial in five cities in Canada	Mean: no head injury group/no LOC, 41.0; head injury with LOC, 40.8	Five cities across Canada: Moncton, Montreal, Toronto, Winnipeg, and Vancouver	Rates in this population of TBI with self-reported LOC. Differences in characteristics, and physical/mental health in people who had LOC vs. not.
To et al., 2015	Journal article	Prospective cohort study	Homeless or vulnerably housed individuals in shelters and meal programs	Median: 43	Three Canadian cities: Toronto, Ottawa, and Vancouver	Characterize associations of having a history of TBI and the use of healthcare, and vulnerabilities associated with TBI and homelessness.
Waldmann, 2012	Abstract	Cross-sectional study	Homeless individuals attending healthcare for the homeless clinics (10 sites)	NR	Boston, MA, USA	Examined incidence of TBI in homeless population in USA.

ACE, adverse childhood events; ACTT, assertive community treatment team; ADL, activities of daily living, BD, bipolar disorder, CSH, chronic subdural hematoma, ED, emergency department; LIH, low-income housing; LOC, loss of consciousness; NR, not reported; SRO, single room occupancy, SSD, schizophrenia spectrum disorder; TBI, traumatic brain injury; UD, unipolar depression; USA, United States of America.

The studies included ranges in sample size from 33 to 2732 participants with a median of 229 participants. One study failed to document its sample size.^20^ The duration of homelessness was poorly characterized. Eight studies reported the duration of homelessness. Of the studies that reported the mean duration of homelessness, the range was 4.4 to 14 years. Of the studies that reported a median range, it was 18 months to 9 years.

The rate of TBI in the studies ranged from 9.2 to 90%, with a median rate of 60.8%. One study focused only on patients with TBI.^20^ North America study locations were: 7 studies in the United States and 13 studies in Canada.

### Primary outcome: The determinants of health related to TBI in the homeless population

The following sub-sections will outline the main results for the association between Health Canada and CDC defined determinants of health. Tabulated and detailed results from the individual studies can be found in Tables 2, 3, and 4. Table 2 provides a tabulated overview of which determinants of health are mentioned in each individual study, whereas Tables 3 and 4 provide a detailed tabulation of the documented associations and data for the secondary outcomes, for those studies without a non-TBI comparator group and those with a non-TBI comparator group, respectively. Of note, the determinant of “income and social status/economic stability” was not assessed in this scoping review, because by default those experiencing homelessness constitute the financially infirm and individuals of poor social status. Similarly, the social determinant of “biology and genetic endowment” will not be commented on in the sub-sections below, as it was not discussed in any of the included studies within this review. Secondary outcomes can be found detailed in a separate subsection below.

**Table 2.** Documentation of Health Canada or CDC Defined Determinants of Health in Individual Studies

Author	Employment and working conditions	Education and literacy	Childhood experiences	Physical environments	Social support and coping skills	Healthy behaviors	Access to healthcare	Biology and genetic endowment	Gender	Culture
Bacciardi et al., 2017	Y	Y	N	N	N	Y	N	N	Y	Y
Barnes et al., 2015	N	N	N	Y	N	Y	N	N	Y	N
Bymaster et al., 2017	Y	Y	Y	Y	N	Y	N	N	Y	N
Cusimano et al., 2018	N	N	Y	Y	N	Y	N	N	N	N
Gargaro et al., 2016	N	Y	N	Y	Y	Y	N	N	Y	N
Gonzalez et al., 2001	N	Y	N	N	N	Y	N	N	Y	Y
Harris et al., 2015	Y	Y	N	N	N	Y	N	N	Y	N
Hwang et al., 2008	N	Y	N	N	N	Y	N	N	Y	Y
Kozloff et al., 2016	Y	Y	Y	Y	N	Y	Y	N	Y	N
Mackelprang et al., 2014	Y	Y	Y	N	Y	Y	N	N	Y	Y
Mejia-Lancheros et al., 2020	N	Y	N	Y	N	Y	N	N	Y	Y
Nikoo et al., 2017	N	Y	N	N	N	Y	N	N	Y	Y
Panenka et al., 2015	N	N	N	Y	N	Y	N	N	Y	N
Solliday-McRoy et al., 2004	Y	Y	N	N	N	Y	N	N	N	Y
Song et al., 2018	N	N	Y	N	N	Y	N	N	Y	N
Svoboda et al., 2014	N	Y	N	N	N	Y	N	N	N	N
Topolovec-Vranic et al., 2014	N	Y	Y	Y	N	Y	N	N	N	N
Topolovec-Vranic et al., 2017	N	Y	N	Y	N	Y	Y	N	Y	N
To et al., 2015	Y	Y	N	N	N	Y	Y	N	Y	Y
Waldmann, 2012	N	N	Y	Y	N	Y	N	N	N	N

CDC, Centers for Disease Control and Prevention; N, not mentioned; Y, yes: some documentation.

**Table 3.** Primary and Secondary Outcomes of Systematic Review: Studies with No Comparator Group

Author	Sample size	Ethical approval	Rate of TBI in study	Injury preceded homelessness	Social determinants discussed	Mental health issues	Sub-population identified
Bacciardi et al., 2017	*N* = 489	Informed consent and REB approval	56.6%	NR	-Male: 71.9% -Unemployment: 95%, -Low educational level: 55.8% -Indigenous: 14.7% -Substance use (healthy behaviors)	Study focused on groups of people with different mental illnesses	BD
Barnes et al., 2015	*N* = 229	Informed consent	90.4%	91.8%: yes	-Male: 96.1% -Moderate/severe TBI post-homeless due to assaults: 23.3%	NR	Veterans
Bymaster et al., 2017	*N* = 127	Informed consent and IRB approval	76%	NR	-Male: 68% -No HS diploma: 24% -Unemployed: 58% -Sustained TBI under age 18: 55.2%, 93.7% -Reported at least 1 ACE -Substance use, alcoholic (self-reported): 59% -Drug use:88%	80% reported they had a mental health disorder, not specified	NR
Cusimano et al., 2018	*N* = 33	No documentation	76%	76%: yes	-ACEs -Assaults in homeless shelter (physical environment)	NR	NR
Gonzalez et al., 2001	*N* = 60	No documentation	38%: head injury	NR	-Male: 60%, -African-American: 56%, -Some type of substance use: 66.7% -Education level: 11.7 years	98.3% of study participants had some type of mental health diagnosis	African-Americans
Harris et al., 2015	NR	No documentation	100% (6% of total TBI/polytrauma patients)	NR	-Female: 100% -Substance use: 24% -Unemployed: 42% -Higher levels of education vs. comparative cohort	Females diagnosed more often than general polytrauma cohort with depression (69% vs. 33%)	NR
Kozloff et al., 2016	*N* = 164	Informed consent and REB approval	61%	NR	-Male: 61% -Indigenous: 27% -Ethnoracial minority:34% -No HS diploma: 76% Unemployed: 96% -Reported unmet healthcare needs: 50% -Arrested in past 6 months: 37%-Drug/Alcohol/Any substance use disorder: 66%, 51%, 74%, respectively -ACE: 87%, -Any assault in past 6 months: 58% (physical environment)	Any mental disorder in study sample ranged from 16% to 51%	Indigenous and ethnoracial minorities
Mejia-Lancheros et al., 2020	*N* = 381	Informed consent and REB approval	9.2%	NR	-Male:68% -Education: 64.9% up to HS -Alcohol/Drug use: 44.6%, 48.8%, respectively -Physical environment: HF group had lower % of violence related TBI than TAU (6.9% vs. 12.3%) -Culture: non-Caucasian ethnoracial background: 57.2%	Study inclusion required having a diagnosable mental illness	Non-Caucasian ethnoracial group
Panenka et al., 2015	*N* = 297	No documentation	Definitive number of 37%; potentially up to 62%	NR	-Male: 81.7% -Alcohol dependence in TBI cohort: *p* = 0.012 -Charged with a criminal offense (p = 0.014) -Assault most common mechanism of injury for TBI	TBI cohort reported more diagnosis of bipolar 1 disorder (*p* = 0.047)	NR
Solliday-McRoy et al., 2004	*N* = 90	Verbal consent obtained	48%	NR	-African American: 81% -Education: 65% HS or less -Unemployed: 68% -Substance abuse/dependency: 93%	50% had some mental health problem	African-Americans
Svoboda et al., 2014	*N* = 170	Informed consent and REB approval	23%^*^	NR	-TBI was greater in alcohol dependent cohort -Education: 63% had vocational training/college and above -Alcohol dependence: 46% total study population	24% of study participants reported a mental health problem	NR
Waldmann, 2012	*N* = 190	No documentation	68%	^**^over 25%	-Reported using drugs or alcohol when the TBI occurred: 5.7% -TBI occurred before age of 10: 25% -Assault was most common cause of TBI -Higher rates of criminal convictions	Statistically significant differences (higher rates) in mental illness	Those with TBI were more likely to be veterans

^*^ Study reported head injury and not all head injury may be TBI, may be soft tissue injury; ^**^no further data, not specific data (abstract only).

ACE, adverse childhood event; BD, bipolar disorder; CI ,confidence interval; HS, high-school; IRB, institutional review board; NR, not reported; OR, odds ratio; REB, research ethics board; TBI, traumatic brain injury.

**Table 4.** Primary and Secondary Outcomes of Systematic Review: Studies with a Non-TBI Comparator Group

Author	Sample size; rate of TBI in study	Ethical approval	Injury preceded homelessness	Sub-population identified	Determinants discussed: TBI cohort	Determinants discussed: Non-TBI cohort	Conclusion
Gargaro et al., 2016	*N* = 63; 56%	REB approval; unclear if informed consent obtained	NR	NR	-Male: 69% (total study participants) -Most common cause of TBI: assaults, 53% (total) -No HS diploma: 33% (total) -Alcohol use: 46% -Alcohol use to intoxication: 19% -Cocaine: 21% -Problems with: -mother, 65% - father, 74% - siblings, 67% (social support)	-Alcohol use: 14% -Alcohol use to intoxication: 7% -Cocaine use: 14% -Problems with: -mother, 33% -father, 15% -siblings, 17%	-Substance use greater in TBI group -Problems with loved ones higher in TBI group
Hwang et al., 2008	*N* = 904; 52.5%	Informed consent and REB approval	70%: yes 22%: after	NR	-Male: 73% -Some HS or less: 53% -Alcohol problems in past 30 days: 42% -Drug problems in past 30 days: 57%	-Male: 59%, -Some HS or less: 52% -Alcohol or drug problems in past 30 days: 28% -Drug problems in past 30 days: 40%	-Percentage of men higher in TBI cohort -Lower educational achievement in TBI cohort -Greater percentage of alcohol and drug problems in TBI cohort
Mackelprang et al., 2014	*N* = 2732; 43%	Informed consent and REB approval	51%: yes	Greater number of bisexuals in TBI cohort (prevalence ratio: 1.26; 95% CI: 1.00, 1.59)	-Female: 54.2% -Male: 45.4% -Greater number of gay/lesbian/bisexual in TBI cohort -Lower education than expected for age: 43.4% -History of foster care: 37.5% -Substance use was higher in all categories (alcohol, marijuana, crack or cocaine, inhalants, methamphetamine, hallucinogens) -History of trauma greater in all categories -Lifetime suicidal behavior: 69.1% (coping skills)	-Female: 70.2% -Male: 29.3% -Lower education than expected for age: 37.3% -History of foster care: 27.6% -Substance use lower in all categories (alcohol, marijuana, crack or cocaine, inhalants, methamphetamine, hallucinogens) -History of trauma: lower in all categories -Lifetime suicidal behavior: 60.3%	-Greater number of females compared with males suffering TBI -Lower education than expected for age in TBI cohort -Greater percentages of history of foster care, history of trauma, and all categories of substance use in TBI cohort -Greater percentage of lifetime suicidal behavior in TBI cohort
Nikoo et al., 2017	*N* = 1190; 60.8%	Informed consent and REB approval	Only incident TBI post-baseline interviews: 33.5%	Indigenous: second largest ethnic group besides Caucasian to suffer TBI	-Male: 62.1-66.3% -Indigenous: 20.3%-22.9% -Low educational achievement: 42.2%-48.3% -Problematic alcohol use: 23.5%-26.2% -Problematic drug use: 31.4%-38.5%	-Male: 65.5-66.7% -Indigenous: 17.3%-18.4% -Low educational achievement: 43.8%-45.6% -Problematic alcohol use: 10.5%-12.1% -Problematic drug use: 17.6%-22.8%	-Greater percentage of males in both TBI and non-TBI compared with females -Indigenous ethnicity higher than any other ethnicity other than Caucasian -Greater percentage of problematic alcohol and drug use in TBI cohort
Song et al., 2018	*N* = 500; 63.6%	Informed consent and REB approval	NR	NR	-Male: 61.2% -Childhood trauma-91% -Drug dependence: 90% -Alcohol dependence: 73.1%	-Male: 57.7% -Childhood trauma: 81.8% -Drug dependence: 85.7% -Alcohol dependence: 74.5%	-Greater percentage of males in TBI cohort -Higher percentage of drug dependence in TBI cohort -Greater percentage of alcohol dependence in non-TBI cohort
Topolovec-Vranic et al., 2014	*N* = 111; 45%	Informed consent and REB approval	87%: yes	NR	-Education: some HS or less: 51% -History of arrest: 86% -History of substance abuse: 68% -Parental substance abuse: 55% -TBI cohort reported assaults were mechanism of injury: 66%	-Education: some HS or less, 38% -History of arrest: 63% -History of substance abuse: 60% -Parental substance abuse: 35%	-Greater percentage of lower educational achievement, history of arrest, history of substance abuse, and parental substance abuse in TBI cohort
Topolovec-Vranic et al., 2017	*N* = 2088; 66% (with LOC: 53%)	Informed consent and REB approval	NR	Aboriginal (Indigenous) 28.9% TBI: OR 2.25 (1.78-2.84) 95% CI	-Male: 71% -Less than high school education: 59% -Aboriginal or ethnoracial: 28.9% and 18.8% -Substance dependence or abuse: 59.4% -Alcohol dependence or abuse: 53.1% -Felt needed health care but didn't receive it: 56.7% -Contact with criminal justice system in past 6 mos:40% -Winnipeg site: 31.7%	-Male: 63.4% -Less than HS education: 51.3% -Aboriginal or ethnoracial ethnicity: 13.5% and 31.2% -Substance dependence or abuse: 45.8% -Alcohol dependence or abuse:35.2% -Felt needed healthcare but didn't receive it: 35.2% -Contact with criminal justice system in past 6 months:30.7%	-Greater percentage of males, lower educational achievement, aboriginal, substance/alcohol dependence or abuse, felt needed healthcare but didn't receive it, and contact with criminal justice system in past 6 months in the TBI cohort
To et al., 2015	*N* = 1181; 61%	Informed consent and REB approval	NR	Indigenous-greater number in this ethnic cohort than any other besides Caucasian	-Male: 71.3% -Indigenous: 18.8% -Some high school education: 43.8% -Unemployed in past 12 months: 58% -Drug abuse screening test moderate, substantial and severe: 60.1% -Harmful/hazardous drinking: 42% -No primary healthcare provider: 39.7%	-Male:58.3% -Indigenous: 16% -Some HS education: 45.9% -Unemployed in past 12 months: 63.7% -Drug abuse screening test moderate, substantial, and severe: 43.2% -Harmful/Hazardous drinking: 30.8% -No primary healthcare provider: 38.8%	-Greater percentage of males, Indigenous ethnicity, drug abuse, harmful/hazardous drinking, and no primary health care provider in the TBI cohort. -Greater percentage of low educational achievement and unemployment in past 12 months in non-TBI cohort

ACE, adverse childhood event; CI, confidence interval; HS, high-school; NR, not reported; OR, odds ratio, REB, research ethics board, TAU, treatment as usual; TBI, traumatic brain injury.

#### Employment and working conditions

Employment and working conditions as a health determinant refers to an individual's level of employment, frequency of work, compensation for work, and the conditions in which they work as this relates to development of disease. Seven studies documented employment and working conditions.^20–26^ Of the seven, unemployment rates of the study participants were discussed. The unemployment rates ranged from 42% to 97.7% across the studies. The study that reported a 97.7% unemployment rate consisted of participants with various mental illnesses and their associations with TBI. Of these studies, five had no comparator group^20–23,26^ and two had a non-TBI comparator group.^24,25^

#### Education and literacy

Education and literacy as a determinant of health refers to the level of academic attainment of an individual and its impact on their social stability and development of disease. The health determinant education and literacy was examined in 15 of the 20 studies.^20–34^ In 10 of these 15 studies, the majority (over 50%) of the study sample reported their maximum educational achievement being high school (HS) completion or equivalent. Of these studies, 8 had no comparator group^20–23,26,30,33,34^ and 7 had a non-TBI comparator group.^24,25,27–29,31,32^

#### Childhood experiences

Childhood experiences as a determinant of health refers to an individual's childhood exposures and the impact on health and disease in adulthood. The determinant of childhood experiences was discussed in 7 studies.^22–24,31,35–37^ Adverse childhood experiences (ACEs) refers to physical, emotional, sexual abuse; emotional and physical neglect; having a family member who abused alcohol or drugs; and being in foster care. Of these 7 studies, it was reported that 23.4–93.7% of the participants suffered some type of ACE. In the studies that reported the differences of homeless individuals with or without TBI, the TBI groups reported higher percentages of participants suffering any type of ACE.^24,31,36^

#### Physical environments

Physical environment as a determinant of health refers to the local and regional shelter and social environment in which the individual finds themselves. There were 10 studies that commented on the determinant of physical environment.^22,23,27,31,32,34,35,37–39^ The majority described homelessness as a risk factor of sustaining a TBI or further TBIs due to the vulnerability that homelessness places on an individual. Further, the most frequent mechanism of injury was assault in 8 of the 10 studies. One study did not discuss the most common mechanism of injury and only stated that in the study, precariously housed individuals were more likely to suffer a TBI with loss of consciousness (LOC) than those suffering from absolute homelessness.^32^ Of these studies, 7 had no TBI comparator group^22,23,34,35,37–39^ and 3 had a comparator cohort.^27,31,32^

#### Social support and coping skills

Social supports and coping skills were not well examined in the studies in the review. Social support and coping skills as a determinant of health refers to the surrounding network of human support and the individual's specific coping skills as they both relate to health and disease. Only two studies commented on social supports and coping skills and both studies included a TBI and non-TBI comparator group.^24,27^ Gargaro and Gerber^27^ reported, among the TBI cohort, 65–74% had problems with their immediate family and close friends compared with 9–33% in the non-TBI cohort. Mackelprang and colleagues^24^ discussed an association of suicidality after suffering a TBI, in relation to coping abilities. However, this was only inferred and not directly assessed in the study.^24^ The paucity of the literature was surprising as the direct relationship of coping skills and homelessness is apparent in many studies discussing past traumatic experiences.^40,41^

#### Healthy behaviors

Healthy behaviors as a health determinant refer to an individual's personal diet, activity and recreational behaviors, and their impact on health and disease. All studies discussed risk taking and/or unhealthy behaviors in relation to TBI and experiencing homelessness.^20–39^ Of the included studies, 8 reported a non-TBI comparator group^24,25,27–29,31,32,36^ and 12 did not have a comparator group.^20–23,26,30,33–35,37–39^ All studies discussed alcohol and/or drug use or dependence behaviors in relation to TBI and homelessness. Of the studies that differentiated the TBI cohort from individuals without TBI, the range of alcohol use/dependence was 16–73% for the TBI cohorts, whereas drug use/dependence ranged from 9% to 90%. Additionally, 5 studies examined criminalistic behavior.^25,31,32,37,39^ They discussed that individuals with a TBI had higher rates of criminal offences compared with those without a TBI.

#### Access to healthcare

Access to healthcare as a determinant of health refers to an individual's physical access and the level of care available, as it related to the development of disease. The reporting of the determinant “access to healthcare” was minimal. This could possibly be because most of the studies were completed in Canada where universal healthcare access is available to all residents. Three studies explored access to healthcare in relation to TBI and experiencing homelessness.^23,25,32^ Of the three studies, two had a non-TBI comparator group^25,32^ and one did not.^23^ Kozloff and associates reported in their study that only 49% of study participants had a regular general practitioner (GP), and half of the participants reported they felt they had a medical need that had not been met in the past 6 months.^23^ Another study reported 56.7% had unmet needs relating to health in the past 6 months.^32^ Finally, one study commented that individuals with a TBI used the emergency department (ED) more frequently than people without a TBI (54.6 % vs. 40.8%, respectively).^25,32^ Although even when healthcare is available, the neuropsychological, physical, and cognitive sequalae that persists after a TBI can leave individuals with many health issues that are not easily diagnosable and/or treatable.^42^

#### Gender

Gender as a health determinant refers to the impact that an individual's gender may play on the various aspects of life and the downstream impact on health and disease. Fifteen studies examined gender as a determinant in relation to experiencing a TBI and homelessness.^20–25,27–29,32–34,36,38,39^ Of the 15 studies, 13 demonstrated larger male representation in the study sample and that males within the studies suffered TBI at a higher rate than females. The range of male participants in the 13 studies was 59.9–96%. The rate of TBI for males versus females was 58–71.3% and 27.2–42%, respectively.^25,28,32,36^ One study had a larger female participant number (63.3%) as well as a higher number of females suffering a TBI than males (54% vs. 45.4%).^24^ One study of the 15 had an all-female study sample.^20^ Of these studies, 7 had a non-TBI comparator group^24,25,27–29,32,36^ and 8 did not include a comparator group.^20–23,33,34,38,39^

#### Culture

Culture as a determinant of health refers to an individual's cultural and ethnic background as it relates to the development of disease. Culture was not emphasized as a significant health determinant in most of the articles in this review. However, there were noteworthy examples of culture and its effect on certain sub-populations experiencing homelessness with TBI. Nine studies commented on culture other than Caucasian ethnicity.^21,24–26,28,29,32–34^ One study described 14.7% of their participants were of Indigenous ethnicity.^21^ One study commented that TBI was less common in the Black or African participants compared with the Caucasian participants.^24^ Comparatively, two studies discussed a higher African-American number of participants.^26,33^ Two studies reported that other than Caucasians, the Indigenous population had the highest rates of TBI in their respective studies.^25,29^ Finally, one study reported the Indigenous population had the second highest TBI prevalence after Caucasians (18.8% and 67.5%, respectively). At one site in this study (Winnipeg), the highest proportion of TBI was found in Indigenous people (70%).^32^ This is of note, as the authors described that the Indigenous population reported TBI with LOC twice as much as the Caucasian group, which indicates the severity of TBI is higher in the Indigenous population. Prior studies have also reported these findings in Indigenous cohorts,^7,43^ as well as sustaining a TBI from a violent act and suffering high rates of substance abuse.^44^ These factors alone, or combined, predispose this group to both TBI and homelessness. Of these studies, five included a non-TBI comparator group^24,25,28,29,32^ and four did not included a comparator group.^21,26,33,34^

### Secondary outcomes: Temporality of TBI, repeat TBI episodes, and sub-populations

#### Temporality of TBI with homelessness and repeat TBI episodes

Of the 20 studies, 5 studies discussed the temporality of suffering a TBI prior to becoming homeless.^24,28,31,35,38^ One study discussed the incidence TBI after the baseline interviews.^29^ Two studies discussed participants experiencing TBI as children; however, they did not specify whether the TBI happened prior to becoming homeless.^22,37^ Of the 5 studies that reported temporality of TBI in relation to homelessness, the majority of the participants suffered their first TBI before becoming homeless. The range of respondents who reported suffering a TBI prior to homelessness was 51–92%.

In contrast, the variance of TBI incidence rates while homeless in the sample homeless populations across North America ranged from 23% to 90.4% in this systematic review. Three of the five studies that commented on the temporal relationship of TBI and homelessness described the incidence of TBI while the study participants were homeless (during the first year or at the age they became homeless?) and after becoming homeless. Experiencing a TBI during the first year of homelessness ranged from 7% to 11.1%.^24,28,38^ The range of participants experiencing TBI after becoming homeless was 22–43.5%.^28,38^

Although there were determinants of health associated with suffering repeat TBI episodes while homeless, the causal factors or interrelation between them are unclear based on current literature included in this systematic review. Six studies discussed at least one determinant of health related to sustaining multiple TBIs.^27–30,34,38^ In the six studies, the determinants discussed in relation to suffering multiple TBIs were assault (physical environments), and alcohol and drug problems (healthy behaviors). Moreover, a few studies discussed the impact that suffering a TBI has on sustaining a TBI in the future. Nikoo and colleagues stated that having a TBI is associated with twice the risk of further TBIs at follow-up and having two TBIs is associated with 8 times the risk of having further TBIs.^29^ Two further studies reported the association of having a TBI placing a person at risk for further TBIs.^22,30^

#### Sub-populations

There were sub-populations within the homeless population samples identified in this review as having greater risks for suffering a TBI. Eleven studies identified at least one sub-group in the homeless population at risk for TBI.^21,23–26,29,32–34,37,38^ One study explained that individuals in the sample who had bipolar disorder had a higher incidence of TBI than the other cohorts in the study.^21^ Two studies displayed higher proportions of African-Americans, which therefore would increase the number of African-Americans suffering TBI in their sample by default.^26,33^ This could be explained by the sampling bias of using a convenience sample. One study reported a non-Caucasian ethnoracial category sustaining a greater number of TBIs.^34^ Two studies reported veterans as a sub-population suffering TBI at high rates.^37,38^ Four studies discussed the Indigenous cohorts in their respective studies suffering TBI at the highest rates, other than the Caucasian ethnic population.^23,25,29,32^ One study of importance reported in Indigenous individuals TBI rates with an odds ratio (OR) of 2.25, 95% confidence interval ([CI]: 1.78-2.84) compared with the Caucasian ethnic sample cohort.^32^ Finally, one study reported that being bisexual was associated with a higher rate of TBI.^24^

#### Determinants in studies with TBI and non-TBI comparator groups

Of the 20 studies in the review, 8 reported having a TBI and non-TBI comparator group. In these 8 studies, determinants associated with homelessness and TBI were discussed. Substance use was reported as greater in the TBI cohort in 8 studies.^24,25,27–29,31,32,36^ One study reported higher alcohol use in the non-TBI cohort.^36^ Five studies discussed the greater percentage of men in the TBI cohort.^25,28,29,32,36^ One study reported a greater number of females in the TBI cohort.^24^ Four studies reported a greater percentage of people having lower educational achievements in the TBI cohort.^24,28,31,32^ One study reported lower educational achievements in the non-TBI cohort.^25^ Three studies described Indigenous ethnicity as the second highest in numbers of ethnic groups, after Caucasian, in the TBI cohort.^25,29,32^ Two studies reported the TBI cohort suffered ACEs in greater percentages than the non-TBI cohort.^24,31^ Two studies reported a greater percentage of problems with social support in the TBI cohort.^24,27^ Two studies reported a greater percentage of contact with criminal justice system in the TBI cohort.^31,32^ Two studies reported higher unmet health needs in the TBI cohort.^25,32^ Finally, one study reported a higher number of unemployment in the non-TBI cohort.^25^

## Discussion

This is the first systematically conducted scoping review examining the determinants of health in relation to TBI in those experiencing homelessness in North America. Several determinants were significantly associated with many of the populations represented in the included studies. Of the studies that included a TBI cohort and comparator non-TBI cohort, there were many associated determinants that tended to negatively affect the TBI cohort more often than the non-TBI cohort. For example, having lower educational attainment, higher substance use, suffering ACEs, higher contact with the criminal justice system, a higher number of males suffering TBI, and greater unmet health needs were all discussed with a larger percentage affecting the individuals who suffered TBIs. This distinction between individuals who suffer TBI and those who do not demonstrates the increased vulnerabilities faced by those who sustain a TBI, highlighting potential areas of future research investment. Some general important findings regarding health determinants in North American TBI populations deserve highlighting. However, prior to doing so, it must be re-emphasized that the goal of this work was not to definitively try to answer which specific health determinants are associated/causal with TBI in those suffering from homelessness, as this is not feasible given the current literature, but merely to highlight the heterogeneous and limited literature body on the topic, to spark future directed studies in this area.

### Major health determinants associated with the homeless TBI population

Under the health determinant “healthy behaviors,” substance use (drugs and or alcohol) was noted to have significant associations in those experiencing both homelessness and TBI. Although associations with substance use have been previously documented in the literature in individuals who have suffered a TBI,^45^ it is unclear whether the substance use is a consequence of sustaining a TBI, or related to prior behaviors of the individuals that may place them at a greater risk for suffering a TBI. However, one study that focused specifically on veterans^46^ discussed substance abuse as one of the top causes of becoming homeless in that population. Therefore, excessive substance use is an area that deserves specific attention in future studies on TBI in the North American populations experiencing homelessness. The focus should be on assessing temporality and frequency of abuse as they relate to the incidence of TBI and risk of subsequent repeat episodes.

Aside from health behaviors, the determinant of “education” was examined frequently within the studies in this review. It was common for the individuals experiencing homelessness who have suffered TBI to have a HS diploma or less education. This association could suggest having lower educational attainments may contribute to lower socioeconomic status and possibly homelessness, which as described in this review is associated with TBI. Further, Sharbafshaaer describes the link between education and the degree of cognitive impairment after a TBI.^47^ The level of impairment a person suffers following a TBI is at least to some degree mitigated by the level of education and the cognitive reserve they had prior to their TBI. Pre-injury education levels may dictate the outcome from the rehabilitation phase after TBI. Therefore, if education levels prior to sustaining a TBI were low, then a person's cognitive functioning will lack that protective effect after a TBI, and they may have decreased cognitive reserve.^48^ This can have ramifications for both rates of social independence after TBI and successful engagement in the workforce.

Childhood experiences were another health determinant that appeared in many of the included studies. Discussed in 7 studies, the prevalence of having suffered an ACE was higher in many of the TBI cohorts compared with the participants who had not sustained a TBI. Afifi and associates^49^ explain that approximately one-third of Canadian children suffer some type of abuse (physical, emotional, sexual), maltreatment, or parental neglect, or they witness violence or have a parent with a mental illness. Suffering these types of experiences as a child may have long-lasting effects if coping mechanisms and support are not sufficient. These maladaptive coping mechanisms can lead to substance abuse, and other risk-taking behaviors, which can lead to sustaining a TBI, then becoming homeless and being unable to transition out of being homeless.^50^ Future work focused on counseling services for the homeless populations not only could aid with some of the underlying emotional and psychological trauma from ACE, but also could improve coping skills and focus on healthier behaviors that may lead to reduced TBI incidence in those experiencing homelessness.

Male gender is overrepresented in both those suffering a TBI and those experiencing homelessness.^51,52^ Additionally, males who are homeless and suffering a TBI concomitantly are overrepresented, as observed in this review, as well as in a previous review by Topolovec-Vranic and co-workers.^53^ Further, it is documented that men generally suffer more severe TBI and have a higher mortality rate due to TBI.^54^ As a result of the severity of the TBI, the rehabilitation period is much longer with less likelihood of returning to baseline functionality.^54^ These factors may contribute to the higher prevalence of men experiencing homelessness, and the frequency of repeat TBIs as described by Nikoo and colleagues.^29^ One study in the review discussed the underrepresentation of females in literature related to TBI in management and outcomes related to TBI and trauma.^20^ As previously stated, females tend to suffer less severe TBI, which may lead to less reporting of their injuries. Females also tend to have more social support than men, which could describe the propensity of a lower proportion of females experiencing homelessness.^54,55^

Physical environment as a health determinant was prevalent due to the living situations of those experiencing homelessness. The most prevalent mechanism of injury for sustaining a TBI in those experiencing homelessness was consistently described as assault.^22,25,27,36,38^ As noted in previous studies, this displays the vulnerability that individuals experiencing homelessness face from violence.^56^ Further, unstable housing and homelessness is associated with not only lifetime occurrence of TBI, but also risk of incurring a TBI over the duration of the homelessness period.^29^ This aspect is amenable to intervention, by providing safe places for people to shelter and improved community patrols in areas known to be endemic for homelessness.

### Less frequently described health determinants in homeless with TBI

There were other social determinants discussed that were less significant, including: employment and working conditions, culture, social support, and access to healthcare. As outlined in the subsections of the Results section, these social determinants display limited available literature documenting their association with TBI in North American homeless populations. At this time, these social determinants require future consideration when designing epidemiological studies on those experiencing homelessness and TBI.

### Secondary outcomes of interest

Temporality of TBI and homelessness needs further exploration in prospective studies, as in many of the studies that commented on temporality the majority of TBIs preceded homelessness.^24,28,31,35,37,38^ This relationship is not well understood and is currently poorly characterized when assessing the association to the determinants of health but may suggest suffering a TBI places vulnerable individuals at risk for becoming homeless. Further, of the studies that reported sustaining a TBI before becoming homeless, most were during childhood. This relationship should be explored further in future research studies.

Additionally, having a TBI and being homeless was associated with being at risk for suffering another TBI.^27–30,38^ People sustaining multiple TBIs are at risk for a spectrum of neurodegenerative conditions from cognitive and mood disturbances to chronic traumatic encephalopathy (CTE) and dementia, which carry various consequences relating to the ability to hold employment, maintain support systems, maintain healthy behaviors and coping skills, and carry out regular activities of daily living, which all are detrimental on an individual, social, and economic level.^57,58^

Finally, certain sub-populations were identified as being at greater risk for TBI and homelessness. The veteran population was noted in U.S. studies to account for approximately 12% of the homeless population,^46^ and to display many of the risk factors that predispose individuals to suffer TBI (substance use, prior TBI, mental health issues).

### Limitations

Despite the wealth of information obtained on the determinants of health associated with TBI in those North Americans experiencing homelessness, there are some significant limitations from this study that deserve highlighting.

First, the included studies are heterogeneous in design, sample size, and outcome reporting. Thus, despite having mentioned some Health Canada or CDC defined determinants of health within the body of the results, documenting such determinants in those North Americans experiencing homelessness and TBI was not the purpose or main outcome of these studies. Table 1 highlights the specific study outcomes of the included studies. As such, even though there appears to be a strong link between various health determinants and TBI in those experiencing homelessness, the results of this scoping review should be taken as preliminary, requiring much further prospective evaluation.

Second, the focus of this review was on the North American populations experiencing homelessness only within Canada and the United States. As such, results highlighted in this review related to the association between health determinants and TBI in those experiencing homelessness may not necessarily be extrapolated to other populations.

Third, there was limited documentation of the temporality, repeat TBI episodes, and sub-groups in those North American populations experiencing homelessness and TBI. This requires much further prospective study, as some preliminary associations were highlighted in this review.

Fourth, assessing study validity and bias, there appears to be a moderate degree of concern for confounding bias in study design and analysis. The majority of studies were cross-sectional, observational, and retrospective in nature, which are considered lower-quality studies due to confounding factors, lack of control groups, lack of randomization, blinding for outcome assessments, convenience sampling, informational source bias (recall bias), and potentially observer bias. This is not surprising as the study population makes it difficult to obtain comprehensive and complete data throughout the cross-sectional design. Further, these studies are some of the only studies on TBI in the North American population experiencing homelessness and are preliminary exploratory works in the area. As such, it is natural to not be all-inclusive in the account of confounders. However, given the validity and bias concerns outlined, the results of this review need to be interpreted with caution.

Finally, many of the Canadian studies arose from the same author group and identical study population. As such, even though there were 20 articles included in the final review, many may be reporting the same population under different retrospective study reports. As such, the total number of unique patients reported across all studies is much less than the grand sum across all 20 articles, thus reducing the overall power and translatability of the results to other North American and global populations experiencing homelessness.

## Conclusion

This scoping review highlights significant associations between male gender, poor physical environment, negative personal health behaviors, ACEs, and limited education in those experiencing homelessness and TBI. Our results also suggest that those experiencing homelessness with TBI in North America suffer poorer health and health consequences than those without TBI. Future empirical research on TBI in the North American population experiencing homelessness will need to focus on these health determinants as potential areas for intervention, which may lead to improved outcomes for those experiencing homelessness with TBI, reduce the frequency of TBI, and enhance transition out of the homeless state with gainful reintegration into society.

## Supplementary Material

Supplemental data

## Abbreviations Used

ACTT: assertive community treatment team
ADL: activities of daily living
BD: bipolar disorder
CASP: Critical Appraisal Skills Programme
CDC: Centers for Disease Control and Prevention
CFI: Canada Foundation for Innovation
CI: confidence interval
CIHR: Canadian Institutes of Health Research
CSH: chronic subdural hematoma
CTE: chronic traumatic encephalopathy
ED: emergency department
GP: general practitioner
HS: high school
HUD: U.S. Department of Housing and Urban Development
IRB: institutional review board
LIH: low-income housing
LOC: loss of consciousness
MPI: Manitoba Public Insurance
NIH: National Institutes of Health
NINDS: National Institute of Neurological Disorders and Stroke
NR: not reported
OR: odds ration
PRISMA: Preferred Reporting Items for Systematic Reviews and Meta-Analysis
REB: research ethics board
RIF: University of Manitoba VPRI Research Investment Fund
SRO: single room occupancy
SSD: schizophrenia spectrum disorder
TBI: traumatic brain injury
TAU: treatment as usual
UD: unipolar depression
USA: United States of America
