# Supplementary material for: Health Determinants among North Americans Experiencing Homelessness and Traumatic Brain Injury: A Scoping Review
Source: Neurotrauma Rep. 2021 Aug 16;2(1):303–21. doi: 10.1089/neur.2021.0010 (PMC8655803; doi:10.1089/neur.2021.0010)
Supplement: Supplemental data [file Supp_Data.docx]

Appendices Table of Contents

[Appendix A: PRISMA Checklist 2](#_Toc3630813)

[Appendix B: Sample Search Strategy MEDLINE 5](#_Toc3630814)

[Appendix C: Databases Searched and North American Professional Society Meetings 7](#_Toc3630815)

[Appendix D: Description of Data Extraction Elements 8](#_Toc3630816)

[Appendix E*:* CASP Assessment of Study Validity and Bias 10](#_Toc3630817)

#

# Appendix A: PRISMA Checklist (2020)

| **Section and Topic** | **Item #** | **Checklist item** | **Location where item is reported** |
| --- | --- | --- | --- |
| **TITLE** | | |  |
| Title | 1 | Identify the report as a systematic review. | 1 |
| **ABSTRACT** | | |  |
| Abstract | 2 | See the PRISMA 2020 for Abstracts checklist. | 2 |
| **INTRODUCTION** | | |  |
| Rationale | 3 | Describe the rationale for the review in the context of existing knowledge. | 3-5 |
| Objectives | 4 | Provide an explicit statement of the objective(s) or question(s) the review addresses. | 4,5 |
| **METHODS** | | |  |
| Eligibility criteria | 5 | Specify the inclusion and exclusion criteria for the review and how studies were grouped for the syntheses. | 6,7 |
| Information sources | 6 | Specify all databases, registers, websites, organisations, reference lists and other sources searched or consulted to identify studies. Specify the date when each source was last searched or consulted. | 6 |
| Search strategy | 7 | Present the full search strategies for all databases, registers and websites, including any filters and limits used. | 6, Appendix B |
| Selection process | 8 | Specify the methods used to decide whether a study met the inclusion criteria of the review, including how many reviewers screened each record and each report retrieved, whether they worked independently, and if applicable, details of automation tools used in the process. | 6,7 |
| Data collection process | 9 | Specify the methods used to collect data from reports, including how many reviewers collected data from each report, whether they worked independently, any processes for obtaining or confirming data from study investigators, and if applicable, details of automation tools used in the process. | 6,7 |
| Data items | 10a | List and define all outcomes for which data were sought. Specify whether all results that were compatible with each outcome domain in each study were sought (e.g. for all measures, time points, analyses), and if not, the methods used to decide which results to collect. | 7, Appendix D |
|  | 10b | List and define all other variables for which data were sought (e.g. participant and intervention characteristics, funding sources). Describe any assumptions made about any missing or unclear information. | 7, Appendix D |
| Study risk of bias assessment | 11 | Specify the methods used to assess risk of bias in the included studies, including details of the tool(s) used, how many reviewers assessed each study and whether they worked independently, and if applicable, details of automation tools used in the process. | 8, Appendix E |
| Effect measures | 12 | Specify for each outcome the effect measure(s) (e.g. risk ratio, mean difference) used in the synthesis or presentation of results. | 8 |
| Synthesis methods | 13a | Describe the processes used to decide which studies were eligible for each synthesis (e.g. tabulating the study intervention characteristics and comparing against the planned groups for each synthesis (item #5)). | 5-7 |
|  | 13b | Describe any methods required to prepare the data for presentation or synthesis, such as handling of missing summary statistics, or data conversions. | 5-7 |
|  | 13c | Describe any methods used to tabulate or visually display results of individual studies and syntheses. | 5-7 |
|  | 13d | Describe any methods used to synthesize results and provide a rationale for the choice(s). If meta-analysis was performed, describe the model(s), method(s) to identify the presence and extent of statistical heterogeneity, and software package(s) used. | 8 |
|  | 13e | Describe any methods used to explore possible causes of heterogeneity among study results (e.g. subgroup analysis, meta-regression). | 5-8 |
|  | 13f | Describe any sensitivity analyses conducted to assess robustness of the synthesized results. | 8 |
| Reporting bias assessment | 14 | Describe any methods used to assess risk of bias due to missing results in a synthesis (arising from reporting biases). | 8, Appendix E |
| Certainty assessment | 15 | Describe any methods used to assess certainty (or confidence) in the body of evidence for an outcome. | NA |
| **RESULTS** | | |  |
| Study selection | 16a | Describe the results of the search and selection process, from the number of records identified in the search to the number of studies included in the review, ideally using a flow diagram. | 8,9, Figure 1 |
|  | 16b | Cite studies that might appear to meet the inclusion criteria, but which were excluded, and explain why they were excluded. | 8,9 |
| Study characteristics | 17 | Cite each included study and present its characteristics. | 8-14, Tables 1-4 |
| Risk of bias in studies | 18 | Present assessments of risk of bias for each included study. | 8-14, Tables 1-4 |
| Results of individual studies | 19 | For all outcomes, present, for each study: (a) summary statistics for each group (where appropriate) and (b) an effect estimate and its precision (e.g. confidence/credible interval), ideally using structured tables or plots. | 8-14, Tables 1-4 |
| Results of syntheses | 20a | For each synthesis, briefly summarise the characteristics and risk of bias among contributing studies. | 8-14, Tables 1-4 |
|  | 20b | Present results of all statistical syntheses conducted. If meta-analysis was done, present for each the summary estimate and its precision (e.g. confidence/credible interval) and measures of statistical heterogeneity. If comparing groups, describe the direction of the effect. | 8-14, Tables 1-4 |
|  | 20c | Present results of all investigations of possible causes of heterogeneity among study results. | 8-14, Tables 1-4 |
|  | 20d | Present results of all sensitivity analyses conducted to assess the robustness of the synthesized results. | 8-14, Tables 1-4 |
| Reporting biases | 21 | Present assessments of risk of bias due to missing results (arising from reporting biases) for each synthesis assessed. | Appendix E |
| Certainty of evidence | 22 | Present assessments of certainty (or confidence) in the body of evidence for each outcome assessed. | NA |
| **DISCUSSION** | | |  |
| Discussion | 23a | Provide a general interpretation of the results in the context of other evidence. | 16-20 |
|  | 23b | Discuss any limitations of the evidence included in the review. | 20,21 |
|  | 23c | Discuss any limitations of the review processes used. | 20,21 |
|  | 23d | Discuss implications of the results for practice, policy, and future research. | 16-21 |
| **OTHER INFORMATION** | | |  |
| Registration and protocol | 24a | Provide registration information for the review, including register name and registration number, or state that the review was not registered. | NA |
|  | 24b | Indicate where the review protocol can be accessed, or state that a protocol was not prepared. | NA |
|  | 24c | Describe and explain any amendments to information provided at registration or in the protocol. | NA |
| Support | 25 | Describe sources of financial or non-financial support for the review, and the role of the funders or sponsors in the review. | 21,22 |
| Competing interests | 26 | Declare any competing interests of review authors. | 21 |
| Availability of data, code and other materials | 27 | Report which of the following are publicly available and where they can be found: template data collection forms; data extracted from included studies; data used for all analyses; analytic code; any other materials used in the review. | NA |

*From:*  Page MJ, McKenzie JE, Bossuyt PM, Boutron I, Hoffmann TC, Mulrow CD, et al. The PRISMA 2020 statement: an updated guideline for reporting systematic reviews. BMJ 2021;372:n71. doi: 10.1136/bmj.n71

For more information, visit: <http://www.prisma-statement.org/>

# Appendix B: Sample Search Strategy MEDLINE

|  |  |  |  |  |  |  |
| --- | --- | --- | --- | --- | --- | --- |
|  | 1 | traumatic brain injury.mp. |  |  |  |  |
|  | 2 | traumatic brain injury.tw. |  |  |  |  |
|  | 3 | TBI.mp. |  |  |  |  |
|  | 4 | TBI.tw. |  |  |  |  |
|  | 5 | brain injury.mp. |  |  |  |  |
|  | 6 | brain injury.tw. |  |  |  |  |
|  | 7 | head injury.mp. |  |  |  |  |
|  | 8 | head injury.tw. |  |  |  |  |
|  | 9 | head trauma.mp. |  |  |  |  |
|  | 10 | head trauma.tw. |  |  |  |  |
|  | 11 | cerebral trauma.mp. |  |  |  |  |
|  | 12 | cerebral trauma.tw. |  |  |  |  |
|  | 13 | brain trauma.mp. |  |  |  |  |
|  | 14 | brain trauma.tw. |  |  |  |  |
|  | 15 | cerebral injury.mp. |  |  |  |  |
|  | 16 | cerebral injury.tw. |  |  |  |  |
|  | 17 | concussion.mp. |  |  |  |  |
|  | 18 | concussion.tw. |  |  |  |  |
|  | 19 | homeless.mp. |  |  |  |  |
|  | 20 | homeless.tw. |  |  |  |  |
|  | 21 | rough sleeping.mp. |  |  |  |  |
|  | 22 | rough sleeping.tw. |  |  |  |  |
|  | 23 | displaced.mp. |  |  |  |  |
|  | 24 | displaced.tw. |  |  |  |  |
|  | 25 | street people.mp. |  |  |  |  |
|  | 26 | street people.tw. |  |  |  |  |
|  | 27 | vulnerable people.mp. |  |  |  |  |
|  | 28 | vulnerable people.tw. |  |  |  |  |
|  | 29 | vulnerable population.mp. |  |  |  |  |
|  | 30 | vulnerable population.tw. |  |  |  |  |
|  | 31 | disadvantaged.mp. |  |  |  |  |
|  | 32 | disadvantaged.tw. |  |  |  |  |
|  | 33 | underserved population.mp. |  |  |  |  |
|  | 34 | underserved population.tw. |  |  |  |  |
|  | 35 | underserved people.mp. |  |  |  |  |
|  | 36 | underserved people.tw. |  |  |  |  |
|  | 37 | sensitive population.mp. |  |  |  |  |
|  | 38 | sensitive population.tw. |  |  |  |  |
|  | 39 | 1 or 2 or 3 or 4 or 5 or 6 or 7 or 8 or 9 or 10 or 11 or 12 or 13 or 14 or 15 or 16 or 17 or 18 |  |  |  |  |
|  | 40 | 19 or 20 or 21 or 22 or 23 or 24 or 25 or 26 or 27 or 28 or 29 or 30 or 31 or 32 or 33 or 34 or 35 or 36 or 37 or 38 |  |  |  |  |
|  | 41 | 39 and 40 |  |  |  |  |

# Appendix C: Databases Searched and North American Professional Society Meetings

Databases: BIOSIS, MEDLINE, CINAHL, EMBASE, SCOPUS and Global Health from inception to December 2020

North American meeting proceedings from the last 5 years of pertinent professional societies focused on the care of individuals with traumatic brain injury, were searched for any research that has not reached manuscript form yet.

Canadian Neurosciences Federation (CNSF)

American Association of Neurosurgeons (AANS)

Congress of Neurological Surgeons (CNS)

World Society of Neurological Surgeon (WSNS)

American Association of Neurology (AAN)

American Neurological Association (ANA)

National Neurotrauma Society (NNS)

International Neurotrauma Society (INTS)

Neurocritical Care Society (NCS).

# Appendix D: Description of Data Extraction Elements

**Descriptive study characteristics**

Journal/Abstract-Describes whether the study identified was published as a journal article or abstract

Study Design-The experimental design of the study

Setting & Context-Describes the setting in which the study took place (i.e. Homeless shelter, Primary care clinic, etc.)

Age Range-Describes the ages of the participants in the study

Geographic Location/Participants-Describes the geographical location of the study (i.e. country, state/province, city)

Data Analysis Method-Describes the statistical method used to analyze the study data

Primary/Secondary Outcomes-Describes the primary and secondary objectives of the study

Sample size-Describes the number of people included in the sample population of the study

Ethical Approval-Explains if and what type of ethical approval was obtained for the study

Rate of TBI in Study-Describes the percentage of participants in the study suffered a TBI

Injury preceded homelessness-Describes the percentage of participants which sustained a the TBI before they became homeless

Determinants Discussed-The determinants of health (as outlined by Health Canada and the CDC) which were discussed in the study

Mental Health Issues-The percentage of mental health issues the study sample had

Sub-Population Identified-Describes the sub-populations discussed in the study that were identified suffering TBI at a significant rate compared to the most prevalent population in the study.

**Social Determinants of Health**

Income and social status-Refers to the amount of money a person receives through employment or investments/social assistance and a person’s position in society in reference to economic, power and employment status.

Employment and working conditions-Refers to an individual’s level of employment, frequency of work, compensation for work and the conditions in which they work as this relates to development of disease

Education and literacy- Refers to the level of academic attainment of an individual and its impact on their social stability, and development of disease.

Childhood experiences- Childhood exposures and the impact on health and disease in adulthood.

Physical environments- refers to the local and regional shelter and social environment in which the individual finds themselves, such as neighborhood, community, etc.

Social supports and coping skills-Refers to the surrounding network of human support and the individual’s specific coping skills as they both relate to health and disease.

Healthy behaviors-Refers to an individual’s health practices as it relates to health and disease. For example, regular health checkups, physical activities, healthy diet, unhealthy practices such as smoking, drinking alcohol, substance use, etc.

Access to healthcare- Refers to an individual’s physical access and the level of care available, as it related to the development of disease

Biology and genetic endowment-Refers to an individual’s biological predispositions and genetic influences on health and disease development.

Gender- Refers to the impact that individual gender may play on the various aspects of life and the downstream impact on health and disease

Culture-Refers to an individual’s cultural and ethnic background as it relates to the development of disease

# Appendix E*:* CASP Assessment of Study Validity and Bias

*Of note, all of the included studies for this review were primarily of cross-sectional design. There currently does not exist a separate CASP tool for cross-sectional study design. As such, the CASP tool for observational studies was adapted to the included studies. This CASP checklist appraises the following domains: Validity, selection bias, classification bias, confounding factors, reporting, and other relevant outcomes which could lead to bias. A summary of the overall literature validity and bias can also be found in Appendix E.

CASP Assessment of Study Validity and Bias

| Author | 1. Did the study address  a clearly  focused issue? | 2. Was the cohort recruited in  an acceptable way? | 3. Was the exposure accurately  measured to minimize bias? | 4. Was the outcome accurately  measured to minimize bias? | 5. (a) Have the authors identified  all-important confounding  factors? | 5. b) Have they taken account of  the confounding factors in the  design and/or analysis? | 6. a) Was the follow up of  subjects complete enough? |
| --- | --- | --- | --- | --- | --- | --- | --- |
| Bacciardi et al., 2017 | Y | Y | N/A | Unsure | unsure | Y | N/A |
| Barnes et al., 2015 | Y | Y | N/A | Y | unsure | Unsure-medical data regarding TBI’s not available or present | N/A |
| Bymaster et al., 2017 | Y | Y | N/A | N-convenience sample | N | N | N/A |
| Cusimano et al., 2018 | Y | unsure | N/A | N-doesn’t describe methods (abstract) | N-doesn’t describe methods (abstract) | N-doesn’t describe methods (abstract) | N/A |
| Gargaro et al., 2016 | Y | Y | N/A | Unsure | N | unsure | N/A |
| Gonzalez et al., 2001 | Y | Y | N/A | Unsure | N | N | N/A |
| Harris et al., 2015. | Y | Y | N/A | Y | Unsure-(abstract) | Unsure-(abstract) | N/A |
| Hwang et al., 2008. | Y | Y | N/A | Y-stratified enrollment, random selection | Y | Y | N/A |
| Kozloff et al., 2016 | Y | Y | N/A | Y | N | Y-but also used monetary incentives which could influence participants | Y |
| Mackelprang et al., 2014 | Y | Y | N/A | Y/N | N-retrospective review of previously completed study | Y/N | N/A |
| Mejia-Lancheros et al., 2020 | Y | Y | N/A | Y | Y | Y-Incentives may have influenced participation | N/A |
| Nikoo et al., 2017 | Y | Y | N/A | Y/N | Unsure | Y-but also used monetary incentives which could influence participants | Y |
| Panenka et al., 2015 | Y | Unsure-(abstract) | N/A | Unsure-(abstract) | unsure | unsure | N/A |
| Solliday-McRoy et al., 2004 | Y | Unsure-methods not very descriptive | N/A | N | N | unsure | N/A |
| Song et al., 2018 | Y | Y | N/A | Y-focused, purposive sampling of minority populations | Y | Y | N/A |
| Svoboda et al., 2013 | Y | Y | N/A | Y | Y/N | Y | N/A |
| Topolovec-Vranic et al., 2014 | Y | Y | N/A | Y-random selection, randomization scheme | Y | Y | N/A |
| Topolovec-Vranic et al., 2017 | Y | Y | N/A | Y | Y/N | Y | N/A |
| To et al., 2015 | Y | Y | N/A | Y | Y/N | Y-but also used monetary incentives which could influence participants | Y |
| Waldmann et al., 2012 | Y | Y | N/A | Unsure-(abstract) | Unsure-(abstract) | Unsure-(abstract) | N/A |

| Author | 6. (b) Was the follow up of  subjects long enough? | 7. What are the results of this study? | 8. How precise are the results? | 9. Do you believe the results? | 10. Can the results be applied to  the local population? | 11. Do the results of this study fit  with other available  evidence? | 12. What are the implications of  this study for practice? |
| --- | --- | --- | --- | --- | --- | --- | --- |
| Bacciardi et al., 2017 | N/A | Positive association of BD and TBI-positive association with BD and substance use | p-<0.001, OR-3.28  p-<0.001, OR-3.63 | Y | unsure | Y | Further research needed |
| Barnes et al., 2015 | N/A | Results support possibility of bi-directional relationship between TBI and homelessness | No statistically significant results | Y | N | Y | Further research needed |
| Bymaster et al., 2017 | N/A | High rates of ACE, unstable home environments, and TBI in this population | No statistically significant results | Y | N | Y | Further research needed |
| Cusimano et al., 2018 | N/A | Highlights the high rate of TBI and childhood abuse in the homeless population.  2ndary-Injuries in childhood may be a predictor of aggression in adulthood | Thematic analysis of interviews | Unsure-small sample, qualitative, recall bias | N | Y | Further research into temporality of TBI and homeless. Also, TBI/ACE and aggression in adulthood |
| Gargaro et al., 2016 | N/A | Large portion of clients at this center were positive for TBI | No statistically significant results | Unsure-small sample, recall bias, medical records not available to corroborate TBI dx/severity | N | Y | Further research in screening homeless for TBI needed |
| Gonzalez et al., 2001 | N/A | Significant neuropsychological impairment among population. | Abbreviated Halstead-Reitain Battery test classified a greater number of patients as neuropsychologically impaired compared to other tests, p-<0.001 | Unsure-small sample size, convenience sample, not all confounders considered | N | unsure | Neuropsychological status needs to be considered in homeless population in health treatment and rehabilitation |
| Harris et al., 2015. | N/A | Females represent small numbers in the TBI cohort of veterans. Current data does not wholly represent this population | Female polytrauma victims dx(p <0.05) more often than the general Polytrauma cohort with depression | Y | N-specific sample population | Y | Further research into less represented populations needed |
| Hwang et al., 2008. | N/A | High prevalence of TBI in homeless sample. TBI usually occurred before first episode of homelessness. History of TBI was associated with many adverse health outcomes among homeless people. | Hx of TBI associated with-p-<0.001-seizures, Alcohol/drug problems in past 30 days, poorer mental and physical health, men, | Y | unsure | Y | Further studies into casual pathways of TBI in homeless, Further screening for TBI in homeless |
| Kozloff et al., 2016 | Y | Youth have unique needs and experiences related to homelessness such as higher rates of substance use, lower educational achievement and victimization-high rates of TBI. | P=<0.001-learning problem/disability, infectious disease, has regular medical doctor, age, low educational rates, lifetime duration of homelessness, age at first homelessness | Y | N | Y | Youth have unique needs relating to homelessness that need further investigation |
| Mackelprang et al., 2014 | N/A | High rates of TBI occur in homeless youth. Encourages adverse outcomes such as mental health dx, substance use, suicidality, victimization. | Unknown | unsure | unsure | Y | Further research needed and more screening for TBI in homeless youth/young adults. |
| Mejia-Lancheros et al., 2020 | N/A | Study participants in the Housing first cohort did not significantly reduce an incident physical-violence related TBI from occurring, it did seem to reduce the risk of frequency of these types of TBI’s compared to the treatment as usual group. | Incident violence related TBI: Housing First vs. Treatment as Usual=p=0.072  Number of violence-related TBI: Housing First Group:  Unadjusted incidence rate ratio:: 0.22 (95% CI, 0.06 to 0.78) p=0.02 | Y | unsure | Y | Further research into housing programs for homeless individuals and their implications on social and medical outcomes of individuals. |
| Nikoo et al., 2017 | Y | Individual with lifetime history of TBI, and history of Mental health diagnoses, substance abuse, younger age, and with poorer mental health and residential instability are at greater risk for future TBI’s. | P=<0.01-age,lifetime prevalence of TBI, HX of epilepsy, Hx of mental health dx, problematic alcohol & drug use, | Y | N | Y | Research into support for variables such as residential instability, mental health, substance abuse in relation to TBI needs to be explored |
| Panenka et al., 2015 | N/A | Individuals in precarious and marginalized housing have high rates of TBI and trauma. These individuals also suffer higher comorbidities than non-TBI group. | dizziness  and fainting-p = 0.001, seizures-p = 0.031, and memory complaints-  p = 0.02, alcohol dependence-p = 0.012, bipolar  I disorder-p = 0.047, charged with criminal offence-p=0.014 | Unsure-(abstract) | N | Y | Research into Specialized Brain injury services may benefit precariously housed individuals |
| Solliday-McRoy et al., 2004 | N/A | Study displayed significant rates (80%) of possible cognitive impairment in the sample | Years of education was only significant result related to scores on FSIQ-p<0.001 | Unsure-confounders not taken into consideration, convenience sample | N | Unsure | High variability in cognitive functioning in the sample.  Further research needed. |
| Song et al., 2018 | N/A | Childhood maltreatment was significantly associated with history of TBI among the homeless.  Secondary-reinforces broad clinical consequences from childhood trauma and consideration when treating TBI patients in homeless population. | Childhood maltreatment association with TBI-p=0.0005, adjusted OR-2.26; 95% CI | Y | unsure | Y | Further studies should address temporality of TBI and ACE |
| Svoboda et al., 2013 | N/A | Previous Head injuries are predictive of having a head injury in the future, along with drug dependence and seizures. | P=<0.0001-age (<39 years), single, lifetime hx of seizures, drug dependence, | Unsure-small sample, retrospective data, doesn’t distinguish between head injury and TBI | N | Y | Further research needed |
| Topolovec-Vranic et al., 2014 | N/A | Assault -most common mechanism of injury. Injuries were common prior to homelessness | p-0.001-age, lifetimes hx of seizures, | Y | N | Y | Further research into TBI patients’ interventions for social functioning to prevent homelessness |
| Topolovec-Vranic et al., 2017 | N/A | Obtaining a TBI with LOC was associated with many adverse outcomes-mental health outcomes, substance use disorders, etc. | p-<0.0001-mental health disorders (depression, manic/hypo-manic episode, PTSD, panic disorder, mood disorder with psychotic features, alcohol/substance dependence/abuse, suicidality, migraines, headaches, seizures), felt needed healthcare but didn’t receive it, contact with criminal justice system in past 6 months | Y | N | Y | Research into specialized resources for this population needed as they display multiple co-morbidities and high vulnerability. |
| To et al., 2015 | Y | Homeless individuals with TBI are more likely to use emergency department services vs. GP, be victims of assault and have contact with criminal justice system than non TBI group | p-<0.0001-male, white, Canadian, poorer health, smokers, arrested/incarcerated, assault victim,  p-<0.05-frequent emergency department user | Y | N | Y | Further studies needed with corroboration of medical records. |
| Waldmann et al., 2012 | N/A | TBI is common among patients who are homeless seeking care in these facilities | unknown | Unsure-(abstract) | N | Y | Care providers to the homeless should screen for TBI in everyone. Further research needed for interventions in this population to placate vulnerabilities that could lead to homelessness. |

***Summary of Study Validity and Bias Assessment***

Adapting the CASP tool for observational studies,^1^ individual study validity and bias were assessed. Given there is no CASP tool available for cross-sectional studies, of which all the included studies were, the observational study CASP tool was employed. Twelve questions from this CASP tool were applicable to cross-sectional study designs, including: study focus, recruitment methods, exposure bias, outcome bias, confounding factor identification, confounding factor accountability in analysis, degree of complete follow-up, duration of follow-up, adequacy of results reporting, precision of results, believability of results, applicability of results, corollary with other studies, and implications of results. Appendix E provides a tabulated account of the results for CASP analysis applied to each included study, as well as an explanation of the individual CASP questions applied.

Across all studies the focus and cohort recruitment techniques were clearly described, with recruitment of participants conducted in an acceptable way, in accordance with ethical principles outlined by the World Health Organization.^2^ Similarly, most studies in general demonstrated accurate outcome definition and measurement, with clear, precise (i.e. statistically significant) and believable results that were congruent with other available evidence in the area of TBI in the North American homeless population. All studies were conducted in a preliminary cross-sectional manner, indicating that despite clear and believable results, the main implications of each study indicated the need for future coordinated multi-center research in the area.

However, despite the above-mentioned positive characteristics of the included studies, the majority of the included studies suffered from some major limitations, as defined by the CASP tool. There was poor exposure measurement and accounting for important confounding factors in TBI both in the study design and analysis conducted. Further this, the follow-up period for participants was either incomplete in many studies due to attrition, and the duration of follow-up was poorly documented in most. Given the lack of account for confounding factors the ability to translate the results for most studies to local populations was severely limited.

Overall, in general the body of literature included in this systematic review displays a moderate degree of study limitations and bias. Finally, all included studies documented predominantly positive correlations between various factors and TBI in those North Americans experiencing homelessness, with very few documenting negative, or lack of, associations. This raises the concern about potential publication bias in this area of the literature, focusing only on positive results, suggesting the interpretation of the results of this literature body should occur with caution and some degree of skepticism.

**References:**

1. CASP. (2018). CASP Checklists. CASP - Crit. Apprais. Ski. Programme .

2. World Health Organization. (2018). WHO | Ethical standards and procedures for research with human beings. WHO [cited 2018 Dec 22 ] Available from: https://www.who.int/ethics/research/en/.

3. Barnes, S.M., Russell, L.M., Hostetter, T.A., Forster, J.E., Devore, M.D., and Brenner, L.A. (2015). Characteristics of Traumatic Brain Injuries Sustained Among Veterans Seeking Homeless Services. J. Health Care Poor Underserved 26, 92–105.

4. Cusimano, M.D., Korman, M., Feher, A., Zhang, S., and Hwang, S.W. (2018). Aggression and victimization among homeless persons with a history of traumatic brain injury. J. Neurotrauma 35, A281.

5. Hwang, S.W., Colantonio, A., Chiu, S., Tolomiczenko, G., Kiss, A., Cowan, L., Redelmeier, D.A., and Levinson, W. (2008). The effect of traumatic brain injury on the health of homeless people. CMAJ Can. Med. Assoc. J. 179, 779–784.

6. Mackelprang, J.L., Harpin, S.B., Grubenhoff, J.A., and Rivara, F.P. (2014). Adverse outcomes among homeless adolescents and young adults who report a history of traumatic brain injury. Am. J. Public Health 104, 1986–1992.

7. Topolovec-Vranic, J., Ennis, N., Howatt, M., Ouchterlony, D., Michalak, A., Masanic, C., Colantonio, A., Hwang, S.W., Kontos, P., Stergiopoulos, V., and Cusimano, M.D. (2014). Traumatic brain injury among men in an urban homeless shelter: observational study of rates and mechanisms of injury. CMAJ Open 2, E69–E76.

8. Nikoo, M., Gadermann, A., To, M.J., Krausz, M., Hwang, S.W., and Palepu, A. (2017). Incidence and Associated Risk Factors of Traumatic Brain Injury in a Cohort of Homeless and Vulnerably Housed Adults in 3 Canadian Cities. J. Head Trauma Rehabil. 32.

9. Bymaster, A., Chung, J., Banke, A., Choi, H.J., and Laird, C. (2017). A Pediatric Profile of a Homeless Patient in San Jose, California. J. Health Care Poor Underserved 28, 582–595.

10. Waldmann, C.A. (2012). Traumatic brain injury in homeless patients. J. Gen. Intern. Med. 2, S338.

11. Gargaro, J., and Gerber, G.J. (2016). brain injury in persons with serious mental illness who have a history of chronic homelessness: could this impact how services are delivered? Can. J. Commun. Ment. Health 35, 69–77.

12. Svoboda, T., and Ramsay, J.T. (2014). High rates of head injury among homeless and low-income housed men: a retrospective cohort study. Emerg Med J 31, 571–575.

13. Mejia-Lancheros, C., Lachaud, J., Stergiopoulos, V., Matheson, F.I., Nisenbaum, R., O’Campo, P., and Hwang, S.W. (2020). Effect of Housing First on violence-related traumatic brain injury in adults with experiences of homelessness and mental illness: findings from the At Home/Chez Soi randomised trial, Toronto site. BMJ Open 10, e038443.

14. Bacciardi, S., Maremmani, A.G.I., Nikoo, N., Cambioli, L., Schütz, C., Jang, K., and Krausz, M. (2017). Is bipolar disorder associated with tramautic brain injury in the homeless? Riv. Psichiatr. 52, 40–46.

15. Kozloff, N., Stergiopoulos, V., Adair, C.E., Cheung, A.H., Misir, V., Townley, G., Bourque, J., Krausz, M., and Goering, P. (2016). The Unique Needs of Homeless Youths With Mental Illness: Baseline Findings From a Housing First Trial. Psychiatr. Serv. Wash. DC 67, 1083–1090.

16. Topolovec-Vranic, J., Schuler, A., Gozdzik, A., Somers, J., Bourque, P.-É., Frankish, C.J., Jbilou, J., Pakzad, S., Palma Lazgare, L.I., and Hwang, S.W. (2017). The high burden of traumatic brain injury and comorbidities amongst homeless adults with mental illness. J. Psychiatr. Res. 87, 53–60.

17. To, M.J., O’brien, K., Palepu, A., Hubley, A.M., Farrell, S., Aubry, T., Gogosis, E., Muckle, W., and Hwang, S.W. (2015). Healthcare Utilization, Legal Incidents, and Victimization Following Traumatic Brain Injury in Homeless and Vulnerably Housed Individuals: A Prospective Cohort Study. J. Head Trauma Rehabil. 30, 270–276.

18. Solliday-McRoy, C., Campbell, T.C., Melchert, T.P., Young, T.J., and Cisler, R.A. (2004). Neuropsychological functioning of homeless men. J. Nerv. Ment. Dis. 192, 471–478.

19. Gonzalez, E.A., Dieter, J.N., Natale, R.A., and Tanner, S.L. (2001). Neuropsychological evaluation of higher functioning homeless persons: a comparison of an abbreviated test battery to the mini-mental state exam. J. Nerv. Ment. Dis. 189, 176–181.

20. Song, M.J.Bs. (Hons), Nikoo, M., Choi, F., Schutz, C.G.M., Jang, K., and Krausz, R.M. (2018). Childhood Trauma and Lifetime Traumatic Brain Injury Among Individuals Who Are Homeless. J. Head Trauma Rehabil. 33, 185–190.

21. McKee, A.C., Cantu, R.C., Nowinski, C.J., Hedley-Whyte, E.T., Gavett, B.E., Budson, A.E., Santini, V.E., Lee, H.-S., Kubilus, C.A., and Stern, R.A. (2009). Chronic Traumatic Encephalopathy in Athletes: Progressive Tauopathy following Repetitive Head Injury. J. Neuropathol. Exp. Neurol. 68, 709–735.

22. Vynorius, K.C., Paquin, A.M., and Seichepine, D.R. (2016). Lifetime Multiple Mild Traumatic Brain Injuries Are Associated with Cognitive and Mood Symptoms in Young Healthy College Students. Front. Neurol. 7.

23. Tsai, J., and Rosenheck, R.A. (2015). Risk factors for homelessness among US veterans. Epidemiol. Rev. 37, 177–195.

24. Zuvekas, S.H., and Hill, S.C. (2000). Income and employment among homeless people: the role of mental health, health and substance abuse. J. Ment. Health Policy Econ. 3, 153–163.

25. Poremski, D., Distasio, J., Hwang, S.W., and Latimer, E. (2015). Employment and Income of People Who Experience Mental Illness and Homelessness in a Large Canadian Sample. Can. J. Psychiatry Rev. Can. Psychiatr. 60, 379–385.

26. Dean, P.J.A., O’Neill, D., and Sterr, A. (2012). Post-concussion syndrome: prevalence after mild traumatic brain injury in comparison with a sample without head injury. Brain Inj. 26, 14–26.

27. Lakhani, A., Townsend, C., and Bishara, J. (2017). Traumatic brain injury amongst indigenous people: a systematic review. Brain Inj. 31, 1718–1730.

28. Zeiler, K.J., and Zeiler, F.A. (2017). Social Determinants of Traumatic Brain Injury in the North American Indigenous Population: A Review. Can. J. Neurol. Sci. J. Can. Sci. Neurol. 44, 525–531.

29. Linton, K.F., and Perrin, P.B. (2017). The Differential Impact of Alcohol and Interpersonal Violence on the Severity of Violent Traumatic Brain Injuries among American Indians. Soc. Work Public Health 32, 202–209.

30. Saewyc EM, Skay CL, Pettingell SL, Reis EA, Bearinger L, Resnick M, Murphy A, and Combs L. (2006). Hazards of stigma: the sexual and physical abuse of gay, lesbian, and bisexual adolescents in the United States and Canada. Child Welfare 85, 195–213.

31. Woodhall-Melnik, J., Dunn, J.R., Svenson, S., Patterson, C., and Matheson, F.I. (2018). Men’s experiences of early life trauma and pathways into long-term homelessness. Child Abuse Negl. 80, 216–225.

32. Davies, B.R., and Allen, N.B. (2017). Trauma and homelessness in youth: Psychopathology and intervention. Clin. Psychol. Rev. 54, 17–28.

33. Menon, D.K., and Bryant, C. (2019). Time for change in acquired brain injury. Lancet Neurol. 18, 28.
